# Ubiquitin-binding site 1 of pathogenic ataxin-3 regulates its toxicity in *Drosophila* models of Spinocerebellar Ataxia Type 3

**DOI:** 10.3389/fnins.2022.1112688

**Published:** 2023-01-17

**Authors:** Matthew V. Prifti, Kozeta Libohova, Autumn L. Harris, Wei-Ling Tsou, Sokol V. Todi

**Affiliations:** ^1^Department of Pharmacology, School of Medicine, Wayne State University, Detroit, MI, United States; ^2^Maximizing Access to Research Careers Program, Wayne State University, Detroit, MI, United States; ^3^Department of Neurology, School of Medicine, Wayne State University, Detroit, MI, United States

**Keywords:** ataxia, CAG triplet repeat, *Drosophila*, misfolding and aggregation, neurodegeneration, polyglutamine (polyQ), ubiquitin

## Abstract

Spinocerebellar Ataxia Type 3 (SCA3) is a member of the family of polyglutamine (polyQ) diseases that are caused by anomalous CAG triplet repeat expansions in several genes. SCA3 results from abnormal polyQ expansion in the deubiquitinase (DUB), ataxin-3 (Atxn3). To understand the role of the different domains of mutant Atxn3 on its pathogenicity, with the hope that they can be explored for therapeutic interventions, we have systematically studied their individual and collective effects on its toxicity. One such domain is ubiquitin-binding site 1 (UbS1) on the catalytic domain of Atxn3; UbS1 is necessary for the enzymatic activity of Atxn3. Here, we investigated the importance of UbS1 on the toxicity of pathogenic Atxn3. We generated transgenic *Drosophila melanogaster* lines that express polyQ-expanded Atxn3 with and without a functional UbS1. We found that mutating UbS1 markedly exacerbates the toxicity of pathogenic Atxn3. Additional studies indicated that UbS1 regulates the toxicity of Atxn3 not by affecting its aggregation or sub-cellular localization, but by impacting its role in ubiquitin processing. Our findings provide additional insights into the role of Atxn3’s domains in the pathogenicity of SCA3.

## Introduction

Polyglutamine (polyQ)-expansion diseases are inherited neurodegenerative disorders that result from the abnormal lengthening of a CAG triplet nucleotide repeat in specific genes, which is translated into a polyQ tract in corresponding proteins. Abnormal polyQ expansion leads to disease protein misfolding, formation of proteinaceous aggregates that include the offending protein, cellular malfunction, and death. The polyQ family consists of 9 clinically distinct disorders including Huntington’s Disease (HD), Dentatorubral–pallidoluysian atrophy, Kennedy’s Disease, and Spinocerebellar Ataxia Type (SCA) 1, 2, 3, 6, 7, and 17 (Todi et al., 2007b; Johnson et al., 2022b).

Spinocerebellar Ataxia Type 3 (SCA3, which is also known as Machado-Joseph Disease) is a progressive, dominant ataxia. SCA3 patients experience coordination and balance issues accompanied by neurodegeneration in cerebellar pathways, substantia nigra, cranial motor nerve nuclei, dentate nuclei, and peripheral neurons. These symptoms typically appear in mid-adulthood; however, the time of their onset and severity is influenced by the length of the polyQ expansion within the disease protein, ataxin-3 (Atxn3). Atxn3 is a ubiquitously expressed deubiquitinating enzyme (DUB) that functions by binding poly-ubiquitin chains and cleaving them; it is involved in protein quality control, cytoskeletal regulation, DNA damage control, sensory organ development and function, and more (Matos et al., 2011, 2019; Costa Mdo and Paulson, 2012; Dantuma and Herzog, 2020; Toulis et al., 2020; Johnson et al., 2022b). In mice, Atxn3 is not essential (Schmitt et al., 2007; Switonski et al., 2011; Reina et al., 2012; Zeng et al., 2013).

**FIGURE 1:**
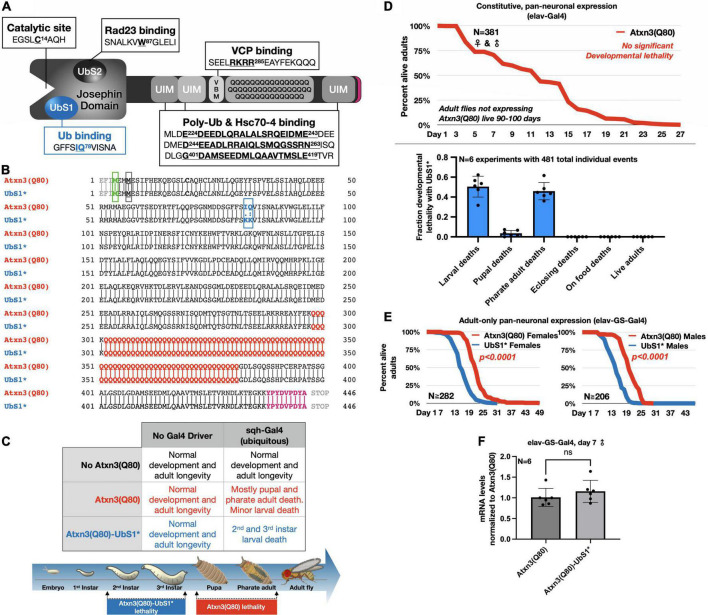
UbS1 mutation exacerbates Atxn3(Q80) phenotypes. **(A)** Diagram of Atxn3 domains. This deubiquitinase (DUB) comprises a catalytic domain known as the Josephin domain and a C-terminal portion that consists of various domains. UbS1, ubiquitin-binding site 1; UbS2, ubiquitin-binding site 2; UIM, ubiquitin-interacting motif; VBM, VCP-binding motif. Boxes associated with domains indicate residues important for interactions. **(B)** Amino acid sequence of Atxn3 proteins used in this study, highlighting first methionine (green); endogenous methionine (black); UbS1 mutation (blue); polyQ tract (red); HA epitope tag (purple). **(C)** Summary of results from crosses leading to the expression of Atxn3 in all tissues (sqh-Gal4). **(D)** Summary of results from crosses leading to the expression of Atxn3 in all neurons (elav-Gal4). **(E)** Summary of results from crosses leading to the expression of Atxn3 in all adult neurons (elav-GS-Gal4). Flies were reared in media without the inducer, RU486, then switched into media with RU486 on the day that they eclosed from their pupal cases. *P*-values are from log-rank tests. **(F)** Quantitative-PCR results from expression of Atxn3 versions in adult fly neurons for 7 days. “ns”: not significant, from Wilcoxon test.

Located on the N-terminal half of Atxn3 (Figure 1A) is the Josephin domain that contains the catalytic site and two ubiquitin-binding sites (UbS1, UbS2) that coordinate spatial interactions between the catalytic groove and ubiquitin (Nicastro et al., 2006, 2009, 2010). UbS2 additionally interacts with the proteasome shuttle protein, Rad23; this interaction controls ataxin-3 stability and toxicity (Nicastro et al., 2006, 2009, 2010; Blount et al., 2014; Tsou et al., 2015b; Sutton et al., 2017). Downstream the Josephin domain resides the C-terminal half of Atxn3, comprising ubiquitin-interacting motifs (UIMs), whose interaction with the chaperone, Hsc70-4 also regulates the toxicity of pathogenic Atxn3; and the VCP-binding domain (VBM), whose interaction with the AAATPase, VCP seeds the aggregation of polyQ-expanded Atxn3 (Boeddrich et al., 2006; Winborn et al., 2008; Ristic et al., 2018; Johnson et al., 2021). Nestled between the VBM and the terminal UIM is the polyQ repeat whose anomalous expansion causes SCA3 (Johnson et al., 2022b). There is also an isoform of Atxn3 that does not contain a terminal, third UIM but instead comprises a degron that expedites the host protein’s degradation (Harris et al., 2010; Johnson et al., 2019; Blount et al., 2020). In this study, we utilize the 3-UIM version of Atxn3 as it is the dominant isoform (Harris et al., 2010).

Over the past several years, our group has dissected the individual and combined effects of the various Atxn3 protein domains to understand their role in SCA3 and to explore their utility as therapeutic targets: The catalytic site, UbS2, the VBM, and the UIMs (Tsou et al., 2013, 2015b; Blount et al., 2014, 2018, 2020; Ristic et al., 2018; Johnson et al., 2019, 2020, 2021, 2022a). The final domain in this series of studies, UbS1, is the focus of this report. Here, to explore the role of UbS1 in Atxn3 pathogenicity we again utilize *Drosophila melanogaster* as the model organism.

As a DUB, recombinant Atxn3 cleaves ubiquitin chains with preference for K63-linked species that are longer than 4 ubiquitin moieties long (Winborn et al., 2008). Detailed structural and biochemical studies of the isolated Josephin domain by the Pastore lab identified UbS1 and UbS2 as important regions for enzymatic activity (Nicastro et al., 2009). UbS1 was shown to be necessary for the activity of Atxn3 *in vitro* (Nicastro et al., 2006, 2009, 2010; Todi et al., 2010), but its importance has not been evaluated *in vivo*. Since mutating the catalytic cysteine renders pathogenic Atxn3 markedly more toxic (Warrick et al., 2005; Tsou et al., 2015b; Sutton et al., 2017), we hypothesize that UbS1 mutation similarly enhances toxicity in *Drosophila*. Indeed, we find that UbS1 mutation (denoted here as UbS1*) causes a marked increase in the toxicity of polyQ-expanded Atxn3 in all tissues and stages tested. However, unlike with the mutation of other domains, UbS1 mutation enhances Atxn3 toxicity without seemingly altering its overall protein levels or aggregation. Instead, based on additional genetics and biochemical evidence, we propose that UbS1 regulates the pathogenicity of Atxn3 by impacting the processing of endogenous ubiquitin species.

## Materials and methods

### Plasmid design

Atxn3(Q80) cDNA sequence was based on the human *ATXN3* sequences used in previous publications (Winborn et al., 2008; Todi et al., 2009, 2010; Harris et al., 2010; Nicastro et al., 2010; Blount et al., 2014, 2020; Tsou et al., 2015b; Costa et al., 2016; Sutton et al., 2017; Ristic et al., 2018; Johnson et al., 2019, 2020, 2021, 2022a). The mutagenesis of Atxn3(Q80)-UbS1*, Atxn3(Q80)-C14*-UbS1* and Atxn3(Q80)-UbS1*-UIM* were carried out by Genscript^1^. The transgenes were subcloned into pWalium-10.moe and fly lines were generated using phiC31 integrase-mediated insertion (Markstein et al., 2008) into attP2 site on chromosome 3 (Duke University Model Systems). The polyQ repeat of Atxn3 comprised an alternating CAGCAA sequence to minimize the possibility of additional toxicity effects from mRNA or unconventional translation processes (Johnson et al., 2022a).

### Antibodies

Anti-HA (rabbit monoclonal C29F4, 1:1000, Cell Signaling Technology); anti-ataxin-3 (mouse monoclonal 1H9, MAB5360, 1:500–1000, Millipore-Sigma); anti-tubulin (mouse monoclonal T5168, 1:10,000, Millipore-Sigma); anti-lamin (mouse monoclonal ADL84.12-5, 1:1000, Developmental Studies Hybridoma Bank); anti-FLAG (rabbit monoclonal D6W5B, 1:500, Cell Signaling Technology); anti-ubiquitin (HRP-conjugated, mouse monoclonal A-5, sc-166553, 1:1000, Santa Cruz Biotechnology); peroxidase-conjugated secondary antibodies: Goat anti-mouse and goat anti-rabbit, 1:5000, were from Jackson ImmunoResearch.

### *Drosophila* husbandry

GMR-Gal4 (#8605) was from the Bloomington *Drosophila* Stock Center. UAS-DnaJ-1 lines were from FlyORF (#002258) and Bloomington *Drosophila* Stock Center (#30553). The following stocks were gifts: sqh-Gal4 (Dr. Daniel P. Kiehart, Duke University), elav-GS-Gal4 (Dr. R. J. Wessells, Wayne State University), elav-Gal4 (Dr. Daniel F. Eberl, University of Iowa). All flies were heterozygous for driver and transgene(s). All transgenic Atxn3 lines used here were generated by us and were reported previously (Tsou et al., 2013, 2015b; Sutton et al., 2017; Johnson et al., 2019, 2020, 2022a; Blount et al., 2020) or are being reported here for the first time, as noted in the results section and figure legends. All crosses were conducted at 25°C in diurnal environments with 12 h light/dark cycles and on conventional cornmeal media. All resulting offspring were maintained in the same conditions, except for crosses using elav-GS-Gal4, where offspring were switched into media containing RU486 soon after emerging as adults to induce the expression of Atxn3 transgenes.

### *Drosophila* longevity

For experiments measuring lifespan, adults were collected daily and secluded into groups of the same size (10 adults per vial) and gender to record deaths, unless otherwise noted. Flies were flipped into new food every other day. The number of adults tracked (N) is noted in figures. In cases where death tracking was focused on the developmental stage, flies were observed from the embryo stage through eclosure or adult death, and deaths at each stage were recorded multiple times each week.

### Western blots and quantification

Western blots were performed with 5 adult flies or 5 dissected fly heads per biological sample (N), depending on the experiment and driver being used. Samples were physically homogenized in boiling lysis buffer [50 mM Tris pH 6.8, 2% SDS, 10% glycerol, 100 mM dithiothreitol (DTT)], sonicated, boiled for 10 min, and centrifuged for 7 min at 13,300 rpm at room temperature. Samples were electrophoresed through pre-cast, 4–20% Tris/Glycine gels (Bio-Rad). ChemiDoc (Bio-Rad) was used to image Western blots, which were then quantified with ImageLab (Bio-Rad). Unless otherwise noted, quantification was done using the volume of whole lanes measuring the levels of each protein of interest and corrected for their respective background: Main band to top of gel for Atxn3 and whole lane for ubiquitin signal. Direct blue stains of total protein were performed by saturating the PVDF membrane for 10 min in 0.008% Direct Blue 71 (Sigma-Aldrich) in 40% ethanol and 10% acetic acid, and then rinsed with a solution of 40% ethanol/10% acetic acid before being air dried and imaged. Direct Blue signal was used as loading control, unless otherwise stated.

### Filter-trap assays

Three whole adult flies were used per sample. Samples were homogenized thoroughly in 200 μL NETN lysis buffer (50 mM Tris, pH 7.5, 150 mM NaCl, 0.5% Nonidet P-40) to which protease inhibitor cocktail (PI; S-8820, Millipore-Sigma) was added. The lysates were then diluted with 200 μL 0.5% SDS in PBS. Diluted lysates were sonicated, centrifuged at 4,500 × *g* for 1 min at room temperature, and then diluted further by combining 100 μL lysate (supernatant only) with 400 μL PBS. 40 μL of this final lysate was added to a Bio-Dot apparatus (Bio-Rad) and vacuumed through a 0.45 μm nitrocellulose membrane (Schleicher and Schuell) that had been pre-incubated in 0.1% SDS in PBS. The membrane was then rinsed with 0.1% SDS/PBS twice, processed for Western blotting, and imaged with ChemiDoc.

### Quantitative PCR

Total RNA was isolated from 5 whole flies or 30 fly heads per sample using TRIzol reagent (Thermo-Fisher Scientific) and treated with TURBO DNAse (Thermo-Fisher Scientific) to remove genomic DNA. cDNA was generated with High-Capacity cDNA Reverse Transcription Kit (Applied Biosystems). Quantitative PCR reactions were carried out on a StepOnePlus system (Applied Biosystems) using Fast SYBR™ Green Master Mix (Applied Biosystems). The PCR cycle threshold (Ct) was used for calculation of the relative mRNA expression level. Rp49 was used as internal control. Primers were: Atxn3-F: 5′-GAATGGCAGAAGGAGGAGTTACTA-3′; Atxn3-R: 5′-GACCCGTCAAGAGAGAATTCAAGT-3′; DnaJ-1-F: 5′-GTACAAGGAGGAGAAGGTGCTG-3′; DnaJ-1-R: 5′-CAGACTGATCTGGGCTGTATACTT-3′; Rp49-F: 5′-AGATCGTGAAGAAGCGCACCAAG-3′; Rp49-R: 5′-CA CCAGGAACTTCTTGAATCCGG-3′.

### Co-immunopurification assays

Ten adult flies or 30 dissected adult fly heads, depending on experiment, were homogenized in 800 μL NETN or 1:1 NETN/PBS lysis buffer supplemented with PI. Samples were incubated at 4°C with agitation for 25 min. Following centrifugation of the incubated samples (5,000 × *g*, 4°C, 10 min), supernatant was combined with bead-bound antibodies and tumbled at 4°C for 4 h. Beads were rinsed five times each with lysis buffer, and bead-bound complexes were eluted by adding Laemmli buffer (Bio-Rad) and boiling.

### Cytoplasmic/nuclear fractionations

Fractionation was performed using the ReadyPrep Protein Extraction Kit (1632089, Bio-Rad) using seven whole flies per group that were lysed in cytoplasmic extraction buffer (Bio-Rad) and processes as delineated by the supplier’s protocols.

### Statistics

Statistical tests were conducted using Prism 9 (GraphPad) and are specified in figure legends. The number of biological replicates is noted on figures and corresponding legends.

## Results

### UbS1 mutation enhances the toxicity of pathogenic Atxn3 in *Drosophila*

To investigate the impact of UbS1 on the pathogenicity of Atxn3, we generated transgenic flies that express pathogenic, full-length, human Atxn3 with 80 polyQ repeats [within human disease range (Johnson et al., 2022b)] with an intact UbS1 domain, as well as one harboring mutations that inactivate it by precluding binding to ubiquitin (Nicastro et al., 2005, 2006, 2009, 2010; Figure 1B). Each transgene is inserted into the same “safe harbor” site on chromosome 3 of *Drosophila*, attP2. A single copy of the transgene is inserted in each case, in the same orientation. The expression system that we utilized to examine the effect of UbS1 mutation is the binary Gal4-UAS system, which enables the expression of targeted transgenes in a specific temporal and spatial manner (Brand and Perrimon, 1993).

Atxn3 is expressed ubiquitously in mammals (Johnson et al., 2022b). We therefore began our studies by examining the effect of pathogenic Atxn3 expression in all fly tissues. We found that ubiquitous expression of pathogenic Atxn3(Q80) with intact UbS1 leads to developmental death in pupal and pharate adult phases; no adults eclose successfully from their pupal cases (Figure 1C). Mutation of UbS1 (UbS1*) markedly enhances this phenotype: Developing flies expressing pathogenic Atxn3(Q80)-UbS1* die as 2^nd^ and 3^rd^ instar larvae (Figure 1C). This enhancement of toxicity was also observed when pathogenic Atxn3 was expressed selectively in neuronal cells, the type of tissue most impacted in SCA3, throughout development. Whereas pathogenic Atxn3 expression pan-neuronally leads to early adult lethality, mutating UbS1 leads to developmental lethality (Figure 1D), enhancing the phenotype. Lastly, since SCA3 is adult-onset, we examined whether expression of pathogenic Atxn3 with an intact or mutated UbS1 is similarly toxic in adult neurons. We found that UbS1 mutation again renders pathogenic Atxn3 more toxic in both male and female adults (Figure 1E); this difference in toxicity is not due to variation in mRNA levels, as the two transgenes are expressed similarly in adult neurons (Figure 1F). We conclude that UbS1 mutation enhances the toxicity of pathogenic Atxn3 in flies.

### UbS1 mutation does not impact Atxn3 aggregation or its sub-cellular distribution

**FIGURE 2:**
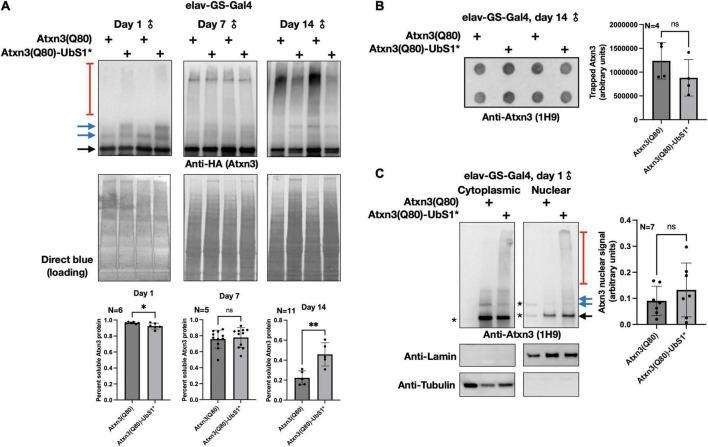
Aggregation of Atxn3(Q80) with intact or mutated UbS1. **(A)** Top: Western blots of Atxn3 versions noted in the figure. Black arrow, main Atxn3 band. Blue arrows, ubiquitinated Atxn3. Red bracketed line, SDS-resistant species of Atxn3. Each lane is an independent repeat. Bottom: Quantification of signal from blots above and other independent repeats. *p < 0.05, ^**^p < 0.01, “ns”, not significant. Statistics: *t*-tests. For quantifications, SDS-soluble species (black and blue arrows) were quantified separately from the SDS-resistant ones (red bracketed lines). **(B)** Left: Filter-trap assay using flies that express Atxn3(Q80) with an intact or mutated UbS1 in adult neurons for 14 days. Right: Quantification of results from the left as well as other, independent repeats. “ns”, not significant. Statistics: *t*-test. **(C)** Left: Results from biochemical separation of Atxn3 protein fractions in cytoplasmic and nuclear compartments when Atxn3 was expressed in adult fly neurons (1 day in RU486). Asterisks, non-specific signal. Black arrow, main Atxn3 band. Blue arrows, ubiquitinated Atxn3. Red bracketed line, SDS-resistant Atxn3. Right: Quantification of the signal from the left and other, independent repeats. “ns”, not significant from *t*-test. The entire Atxn3 signal in each lane was used for quantifications. Tubulin and lamin served as loading controls for their respective fractions.

In our prior investigations into the toxicity of polyQ-expanded Atxn3, we found that increased pathogenicity often correlates with higher aggregation of the SCA3 protein (Tsou et al., 2013, 2015b; Blount et al., 2014; Sutton et al., 2017; Ristic et al., 2018; Johnson et al., 2019, 2020, 2021). Therefore, we examined whether UbS1 mutation impacts the aggregation of Atxn3 with an expanded polyQ repeat. As shown in Figure 2, temporal examination of SDS-resistant species from flies expressing Atxn3(Q80) only in adult neurons does not lead to consistent differences between transgenes with an intact or mutated UbS1, and the overall levels of Atxn3 protein are also comparable between the two versions (Figure 2A; additional examples are in Figures 4–6). Filter-trap assays, used to examine larger aggregates that do not enter SDS-PAGE gels, do not indicate that UbS1-mutated pathogenic Atxn3 is more or less aggregation prone in fly neurons (Figure 2B). Because pathogenic Atxn3 is most toxic when it localizes to the nucleus (Bichelmeier et al., 2007), we also examined whether UbS1 mutation impacts sub-cellular distribution. Based on biochemical cytoplasmic/nuclear separation, this was not the case (Figure 2C). We conclude that UbS1 does not impact the levels, localization or aggregation of Atxn3(Q80) in a manner that correlates with the markedly increased toxicity. We next turned our focus to activities of Atxn3 that may be impacted by this binding domain.

**FIGURE 3:**
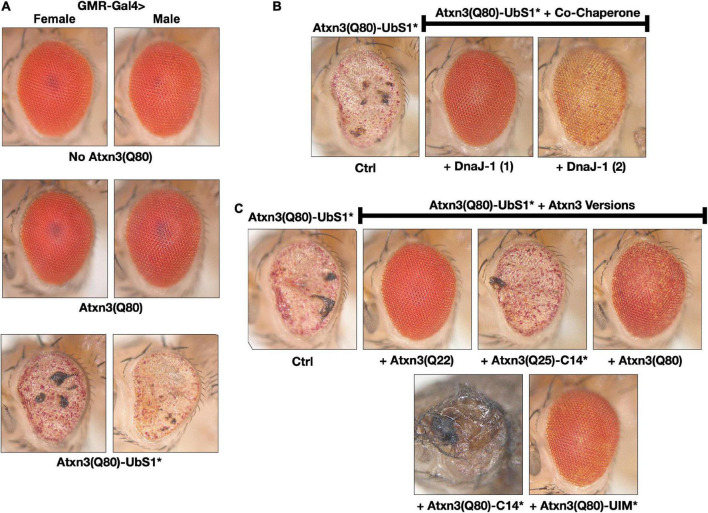
Toxicity from Atxn3(Q80)-UbS1* in fly eyes is modulated by DnaJ-1 and catalytically active Atxn3. **(A–C**) Images of external fly eyes expressing the noted transgenes. All flies were 1 day old. C14*: Mutated catalytic cysteine at position 14 into alanine to render Atxn3 catalytically inactive (Winborn et al., 2008). UIM*: Mutations in each of the ubiquitin-interacting motifs of Atxn3 to disable their interaction with ubiquitin chains (Winborn et al., 2008; Todi et al., 2009). Q(n): Length of the polyQ, where 22 and 25 denote wild-type range and 80 denotes disease range.

**FIGURE 4:**
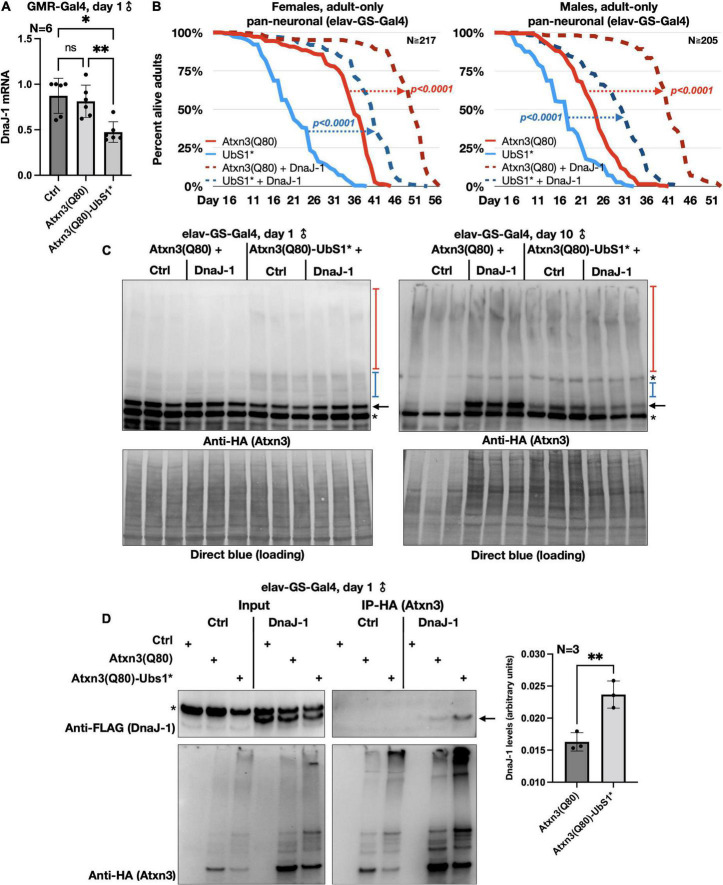
DnaJ-1 suppresses neuronal toxicity from UbS1-mutated Atxn3 and binds it more strongly than Atxn3(Q80) with intact UbS1. **(A)** qRT-PCR from dissected fly heads expressing the noted versions of Atxn3. Ctrl: Gal4 driver on the background line of Atxn3 transgenic flies. *p < 0.05, ^**^p < 0.01, “ns”, not significant. Statistics: One-way ANOVA with Tukey’s *post hoc*. **(B)** Adult fly longevities when Atxn3(Q80) with intact or mutated UbS1 was expressed in adult neurons in the absence or presence of exogenous DnaJ-1. *P*-values are from log-rank tests. **(C)** Western blots from whole flies expressing the noted transgenes in adult neurons for the indicated amounts of time. Black arrow, unmodified Atxn3 band. Blue bracketed bar, ubiquitinated Atxn3 species. Red bracketed bar, SDS-resistant Atxn3. Asterisks, non-specific signal. Ctrl: Flies expressing Atxn3 in the absence of exogenous DnaJ-1. **(D)** Left: Western blots of input and HA-IPs. Black arrow, DnaJ-1 band. Asterisk, non-specific signal. Right: Quantification of blots from the left and other, independent repeats. ^**^p < 0.01 from *t*-test.

**FIGURE 5:**
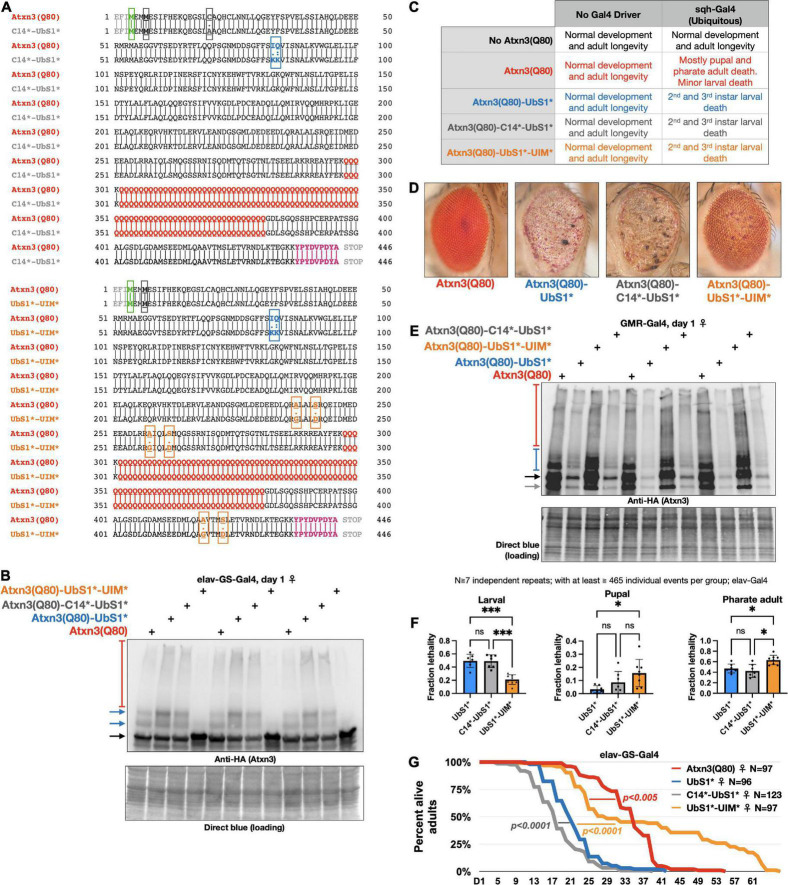
Effect of catalytic cysteine mutation and UIM mutations on toxicity from UbS1*. **(A)** Amino acid sequences of the transgenic Atxn3 expressed in the new lines that we generated. Highlighted: First methionine (green), endogenous methionine (black); C14 mutation (gray); UbS1 mutation (blue); polyQ tract (red); UIM mutations (orange); HA epitope tag (purple). **(B)** Western blots of flies expressing the noted versions of Atxn3 in adult neurons for 1 day. Each lane is an independent repeat. Black arrow, main Atxn3 band. Blue arrows, ubiquitinated Atxn3. Red bracketed bar, SDS-resistant Atxn3. **(C)** Summary of results when the noted versions of Atxn3 are expressed in all tissues. **(D)** Representative photographs of external eyes expressing the noted versions of Atxn3. Flies were 1 day old. **(E)** Western blots from expression of the noted versions of Atxn3 in fly eyes. Flies were 1 day old. Each lane is an independent repeat. Black arrow, main Atxn3 band. Blue bracketed bar, ubiquitinated Atxn3. Red bracketed bar: SDS-resistant species of Atxn3. Gray arrow: Proteolytic fragment we observe sometimes. Reduced Atxn3 signal intensity in the lanes with UbS1* and C14*-UbS1* mutations are from loss of eye mass due to degeneration. **(F)** Summary of results when the noted versions of Atxn3 are expressed in all neurons, throughout development (elav-Gal4). “ns”, not significant. *p < 0.05, ^***^p < 0.001. Statistics: Brown-Forsythe and Welch ANOVA tests. **(G)** Longevity of flies expressing the noted versions of Atxn3(Q80) in all adult neurons (elav-GS-Gal4). *P*-values: log-rank tests.

**FIGURE 6:**
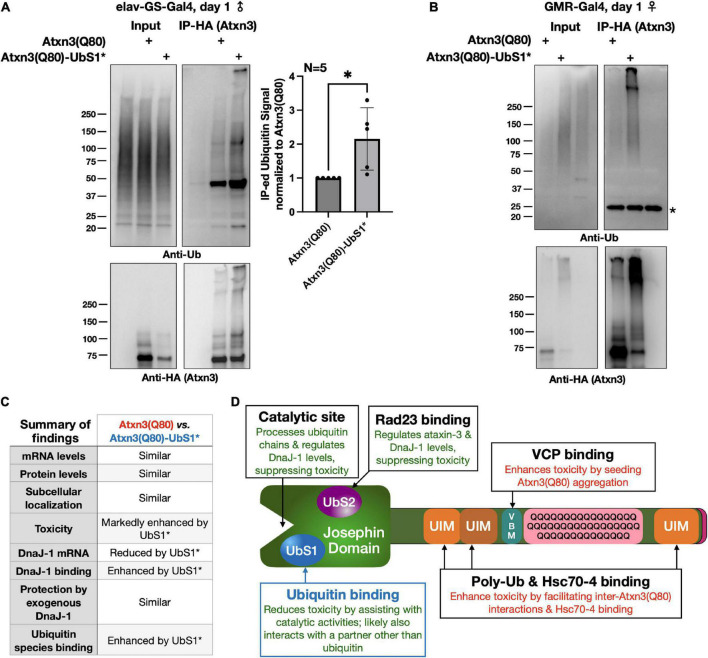
Atxn3(Q80)-UbS1* co-precipitates more ubiquitinated species than Atxn3(Q80). **(A,B)** Western blots of Atxn3(Q80)-HA co-IPs. Ub, ubiquitin; **(A)** Adult neuronal expression; **(B)** Eye expression. Graph: Quantification of results on the left and additional, independent repeats. *p < 0.05, Wilcoxon test. Some of the ubiquitinated species on the anti-ubiquitin blot may include ubiquitinated Atxn3 itself, which is ubiquitinated and interacts with other Atxn3 proteins (Todi et al., 2007a,^2009^, ^2010^; Johnson et al., 2021). **(C)** Summary of the findings presented in this study. **(D)** Graphical summary of the role of various Atxn3 domains, based on our studies in *Drosophila* models of SCA3; references are in main text.

### UbS1 mutation enhances Atxn3 toxicity in fly eyes, revealing potential mechanism of action

Expression of pathogenic Atxn3 with an intact UbS1 in fly eyes does not lead to detectable structural anomalies on day 1 adults, as we showed before (Johnson et al., 2021) and as pictured in Figure 3A; eye degeneration does ensue over time (Johnson et al., 2020, 2021). However, expression of pathogenic Atxn3(Q80)-UbS1* is markedly toxic to fly eyes from day 1 in adults, regardless of sex (Figure 3A), consistent with the increased toxicity that we observed in other tissues because of UbS1 mutation (Figure 1).

To explore which aspects of Atxn3 functions in flies may account for the increased toxicity of UbS1*, we continued using fly eyes (Bonini and Fortini, 2003; Zhang et al., 2010; Casci and Pandey, 2015; Perrimon et al., 2016). Since we did not see a detectable difference between males and females in this phenotype, for the rest of eye-based studies we used males and females interchangeably. We previously reported that wild-type Atxn3 leads to higher levels of the co-chaperone, DnaJ-1 in *Drosophila* at transcript and protein levels in a catalytic activity-dependent manner (Tsou et al., 2015b). This increase in DnaJ-1 levels is neuroprotective against SCA3 and other polyQ models (Tsou et al., 2015a,b). As shown in Figure 3B, over-expression of DnaJ-1 from two different transgenic lines suppresses the UbS1* eye phenotype.

At least in part by increasing DnaJ-1 levels, Atxn3 protects against other polyQ disease proteins in the fly in a catalytic activity-dependent manner (Tsou et al., 2015a,b). As shown in Figure 3C, expression of various forms of Atxn3 that are catalytically active (wild-type, polyQ-expanded, or polyQ-expanded but with mutated UIMs) markedly corrects the UbS1* phenotype. However, catalytically inactive Atxn3 is either unable to suppress UbS1* toxicity [Atxn3 with normal polyQ and mutated catalytic cysteine; C14*(Winborn et al., 2008)] or enhances UbS1* toxicity (polyQ-expanded Atxn3 with mutated C14). These results suggest that a key component of UbS1*-related toxicity is the catalytic activity of Atxn3. This is not entirely surprising, because mutating UbS1 abrogates the DUB activity of Atxn3 *in vitro* (Nicastro et al., 2010). We next explored the role of DnaJ-1 in UbS1*-dependent phenotypes in flies.

### UbS1* leads to lower DnaJ-1 levels, whose overexpression suppresses neuronal toxicity

We began by examining the mRNA levels of DnaJ-1 in flies expressing UbS1-mutated Atxn3. We observed that DnaJ-1 mRNA levels are significantly lower in the presence of Atxn3(Q80)-UbS1* (Figure 4A), similarly to what we reported before for inactive Atxn3 (C14*) with a normal polyQ repeat (Tsou et al., 2015b). (Due to the unavailability of antibodies that reliably detect fly DnaJ-1, we were unable to investigate the endogenous protein for this study). Next, we confirmed that exogenous DnaJ-1 is protective against UbS1-mutated, pathogenic Atxn3 in tissues other than eyes. As shown in Figure 4B, co-expression of DnaJ-1 in adult fly neurons reduces the toxicity of both Atxn3 with a functional UbS1 and with UbS1*, independently of sex (Figure 4B). Concomitant with reduced toxicity, we observed that exogenous DnaJ-1 leads to higher levels of SDS-soluble Atxn3, but does not visibly impact the SDS-soluble/insoluble levels of UbS1-mutated Atxn3 (Figure 4C). Lastly, according to co-immunoprecipitation (co-IP) assays, pathogenic Atxn3 with mutated UbS1 interacts more strongly with exogenous, FLAG-tagged DnaJ-1 compared to pathogenic Atxn3 with intact UbS1. We interpret these data to suggest that UbS1 mutation enhances Atxn3(Q80) toxicity at least in part by compromising endogenous DnaJ-1 expression as well as its downstream functions because of a tighter interaction with the disease protein.

### The role of Atxn3 ubiquitin binding and cleavage on UbS1*-dependent toxicity

As a result of: (1) previously published results that UbS1* renders Atxn3 catalytically dead (Nicastro et al., 2009, 2010), and (2) the current observations that any version of Atxn3 that is catalytically active suppresses toxicity from Atxn3(Q80)-UbS1* (Figure 3C), we examined genetically the impact of the catalytic site and of the UIMs on UbS1*-dependent toxicity. The point we sought to explore was whether Atxn3(Q80)-UbS1* is more toxic because it can bind ubiquitin chains, but it is unable to cleave them thus potentially sequestering various ubiquitinated species and perturbing homeostasis. If that is the case, rendering the catalytic site inactive by mutating the cysteine at position 14 into alanine should not impact the UbS1* phenotype, whereas mutating the UIMs, thus precluding the binding of ubiquitin chains by Atxn3, should ameliorate it.

To explore these possibilities, we generated additional, transgenic fly lines that express Atxn3(Q80)-UbS1* either with a catalytic cysteine mutation (C14*), or with mutated UIMs [(Winborn et al., 2008; Todi et al., 2009) UIM*; Figure 5A]. We confirmed that each transgenic line expresses pathogenic Atxn3 and that the overall protein levels are comparable (Figure 5B; mutation of the UIMs of Atxn3 can reduce the extent of its own ubiquitination in some circumstances Berke et al., 2005; Todi et al., 2007a). With these new lines on hand, we examined the effect of expressing the transgenes everywhere, only in eyes, or only in neuronal tissue.

Ubiquitous expression leads to larval death in every line expressing Atxn3(Q80)-UbS1*, regardless of additional mutations (Figure 5C); we did not observe changes in phenotype with this expression pattern. Expression in fly eyes allowed us to detect clear variation from the toxicity of UbS1* with additional mutations. Mutating C14 in addition to UbS1 leads to an external eye phenotype that resembles UbS1*, whereas mutating the UIMs improves the phenotypes, but does not suppress it to the level of pathogenic Atxn3 without UbS1 mutations (Figures 5D,E shows the relative expression levels of the transgenes in Figure 5D).

We then examined whether the C14 and UIM mutations impact the UbS1* phenotype in all neurons. As we showed in Figure 1, pan-neuronal expression of Atxn3(Q80)-UbS1* during development leads to lethality at larval, pupal, and pharate adult stages. Expression of Atxn3(Q80)-UbS1*-UIM* in all neurons suppresses toxicity compared to the version with intact UIMs (Figure 5F), but this improvement falls short of what we observe with Atxn3(Q80) without UbS1 mutation, which leads to adult flies coming out (Figure 1). Mutating the catalytic cysteine in addition to UbS1 leads to a phenotype similar to the UbS1 mutation alone (Figure 5F).

Lastly, we compared the longevity of flies that expressed UbS1*-containing Atxn3(Q80) without additional mutations, or in addition to C14* or UIM*, only in adult neurons. We observed that mutating the catalytic cysteine slightly but significantly worsens the UbS1* phenotype (Figure 5G). However, UIM* mutations markedly improve the UbS1* longevity phenotype to an extent that is significantly better even when compared to the longevity of adults expressing Atxn3(Q80) without UbS1 mutation (Figure 5G). Collectively, these results indicate that ubiquitin binding and processing both contribute to the toxicity of Atxn3(Q80)-UbS1*: C14 mutation either does not impact phenotype or can worsen it under some circumstances, whereas the UIM* mutations can improve it to varying degrees, depending on the type of tissue and stage of expression.

### Pathogenic Atxn3 with mutated UbS1 interacts more strongly with ubiquitinated species

The reduction in toxicity that we observed when the UIMs of Atxn3(Q80)-UbS1* are disrupted led us to examine whether UbS1-mutated Atxn3(Q80) interacts more strongly with ubiquitinated species in flies. We expressed Atxn3(Q80) with intact or mutated UbS1 in adult fly neurons for 24 h, IP-ed Atxn3, and then examined the levels of ubiquitin species that co-precipitate with it. We observed that UbS1 mutation leads to higher amounts of ubiquitinated species co-IP-ing with Atxn3(Q80)-UbS1* compared to Atxn3(Q80) (Figure 6A). We observed the same pattern when utilizing fly eyes (Figure 6B). We conclude that UbS1 mutation increases the association of pathogenic Atxn3 with ubiquitinated species in *Drosophila*. Alongside the other data presented here (Figure 6C), we propose that UbS1 mutation enhances the toxicity of pathogenic Atxn3 at least in part because of its impaired functions as a DUB (Figure 6D). But, as discussed below, this need not be the only route of action.

## Discussion

Protein-protein interactions play critical roles in the precise symptomatology of polyQ diseases (Johnson et al., 2022b). Toward understanding the role of non-polyQ domains on the toxicity of the SCA3 protein, over the recent years we embarked on systematic studies to dissect the manner in which each of its domains impacts polyQ toxicity (our findings from various publications are summarized in Figure 6D; Blount et al., 2014; Tsou et al., 2015b; Sutton et al., 2017; Ristic et al., 2018; Johnson et al., 2019, 2020, 2021, 2022a). In this study, we investigated UbS1 thereby completing our series of Atxn3 domain examinations from the perspective of *Drosophila melanogaster*. These investigations have collectively increased the understanding of the disease-causing properties of Atxn3 and have identified potential therapeutic points of intervention.

Here, we determined that UbS1 is a key player in the toxicity of pathogenic Atxn3, not dissimilar from the effect of the catalytic site of this DUB—mutation of each domain markedly increases the toxicity of pathogenic Atxn3 in the fruit fly [the data in this report and references (Warrick et al., 2005; Tsou et al., 2013, 2015b; Sutton et al., 2017)]. This outcome was expected, because: (1) we previously demonstrated that UbS1 is important for the catalytic activities of Atxn3 *in vitro* (Nicastro et al., 2010); (2) we and others showed that mutating the catalytic site of Atxn3 renders the pathogenic protein markedly more toxic (Warrick et al., 2005; Tsou et al., 2015b; Sutton et al., 2017); (3) studies from others and us showed that Atxn3 has protective properties in *Drosophila*, which depend on its catalytic activity (Warrick et al., 2005; Tsou et al., 2015b; Sutton et al., 2017); and (4) even the pathogenic version of Atxn3 has protective properties against polyQ-dependent degeneration, as long as it is catalytically active (Figure 3C). Thus, we originally hypothesized that mutating UbS1 would act similarly to mutating the catalytic site, enhancing Atxn3 pathogenicity. Our results in flies generally support this hypothesis.

Why does UbS1 mutation render pathogenic Atxn3 more toxic in *Drosophila*? The mechanism of this enhanced toxicity remains to be determined and will require a deeper understanding of the biological functions of wild-type Atxn3 and how those functions are impacted by polyQ expansions. Those examinations are outside the scope of this study and are confounded by the fact that the precise role of Atxn3 in intact organisms remains unclear. While various studies have provided significant evidence that this DUB is involved in endoplasmic reticulum-associated degradation and other types of protein quality control, DNA damage repair, response to heat stress, transcriptional regulation, sensory organ development and function, synaptic communication, etc. (Costa Mdo and Paulson, 2012; Matos et al., 2019; Herzog et al., 2020; Toulis et al., 2020, 2022), it is also true that mice that lack Atxn3 are generally fine (Schmitt et al., 2007; Reina et al., 2010; Switonski et al., 2011; Zeng et al., 2013). As with other diseases in the polyQ family, more remains to be learned about the SCA3 biology of disease. We hope that the studies that we conducted, including with UbS1, will provide new clues.

What we propose based on this study and the investigations of other domains of Atxn3 (Blount et al., 2014; Tsou et al., 2015b; Sutton et al., 2017; Ristic et al., 2018; Johnson et al., 2019, 2020, 2021, 2022a) is that mutations in UbS1 render this DUB unable to process ubiquitin species—which it binds through the UIMs—due to compromised catalytic activity. As noted above, UbS1 coordinates ubiquitin to allow for proper “attack” of isopeptide bonds by the catalytic site (Nicastro et al., 2009, 2010). In the absence of this interaction, Atxn3 binds but cannot process ubiquitinated species thereby, we posit, sequestrating them. Consequently, UIM mutations alleviate the increased toxicity of UbS1*. We propose that these ubiquitinated species—that have been sequestered—include proteins necessary for cellular biology. Their identity remains unknown at this time and will be the focus of future investigations, but may involve factors important in the transcription of the co-chaperone, DnaJ-1, whose levels are reduced in the presence of UbS1* pathogenic Atxn3 in fly eyes. Additionally, considering: (1) that we observe a stronger interaction of UbS1-mutated Atxn3 with DnaJ-1 compared to Atxn3 with an intact UbS1; and (2) that exogenous DnaJ-1 improves UbS1* pathology, DnaJ-1 may be one factor sequestered by Atxn3(Q80)-UbS1*. (As noted below, future studies will shed light on the interactions and functions of UbS1 by itself and in concert with other ubiquitin binding and cleaving domains of Atxn3). Furthermore, as we reported recently (Johnson et al., 2020), the UIMs of Atxn3 also help it interact with the heat shock protein, Hsc70-4 in *Drosophila* and also help mediate Atxn3-Atxn3 interactions in the fly, which also occur in mammalian cells (Todi et al., 2007a). Thus, some potential substrates of Atxn3 could include heat shock proteins beyond DnaJ-1 as well as other Atxn3 proteins. We predict that complex interactions are at play that collectively lead to the enhanced toxicity of UbS1*, potentially in a tissue-dependent manner.

However, the above is likely not the only mechanism through which UbS1-mutated, pathogenic Atxn3 becomes more toxic. After all, UIM mutations do not fully reverse UbS1*-dependent toxicity in eyes and in developing neurons, and have no detectable modifying effect on UbS1-mutated Atxn3 expressed everywhere. Also, mutation of the catalytic cysteine of Atxn3 enhances UbS1*-dependent toxicity in adult neurons—although it does not noticeably impact it when tested in fly eyes or during development. Perhaps UbS1 also directly binds proteins other than ubiquitin and these interactions further impact Atxn3 toxicity. This idea is supported by the fact that the other ubiquitin-binding site on Atxn3’s catalytic domain, UbS2 directly interacts with both ubiquitin and the proteasome shuttle protein, Rad23; the latter interaction has critical outcomes for the degradation and pathogenicity of Atxn3 (Nicastro et al., 2005, 2009, 2010; Blount et al., 2014; Sutton et al., 2017). This proposed, novel UbS1 interaction may also be tissue-specific and could explain differences in the modulation of toxicity of UbS1* in all tissues vs. eyes vs. neurons by catalytic cysteine and UIM mutations. We will pursue the possibility of UbS1 interactions with non-ubiquitin proteins in the future, with the added hope that this potential interaction may unveil entry points for SCA3 therapy.

To conclude, UbS1 mutations exacerbate the toxicity of Atxn3 in *Drosophila* (Figure 6D), at least in part due to compromised ubiquitin processing by this DUB. Continued interrogation of this disease protein is necessary to understand the biology of SCA3 and to devise therapeutics for it. We believe that this investigation into the pathophysiological properties of polyQ-expanded Atxn3 will prove impactful toward such efforts.

## Data availability statement

The raw data supporting the conclusions of this article will be made available by the authors, without undue reservation.

## Author contributions

MP: conceptualization, data curation, software, formal analysis, funding acquisition, validation, investigation, visualization, methodology, and writing and editing. KL: data curation, formal analysis, supervision, validation, investigation, and visualization. AH: data curation, formal analysis, validation, and visualization. W-LT: conceptualization, data curation, software, validation, investigation, visualization, methodology, and writing and editing. ST: conceptualization, resources, data curation, software, formal analysis, supervision, funding acquisition, validation, investigation, visualization, methodology, and writing and editing. All authors contributed to the article and approved the submitted version.

## Footnotes

1
genscript.com
